# Crystal structure, Hirshfeld surface analysis and density functional theory study of 1-nonyl-3-phenyl­quinoxalin-2-one

**DOI:** 10.1107/S2056989021009737

**Published:** 2021-09-24

**Authors:** Nadeem Abad, Karim Chkirate, Fares Hezam Al-Ostoot, Luc Van Meervelt, Sanae Lahmidi, Souad Ferfra, Youssef Ramli, El Mokhtar Essassi

**Affiliations:** a Laboratory of Heterocyclic Organic Chemistry URAC 21, Pharmacochemistry Competence Center, Av. Ibn Battouta, BP 1014, Faculty of Sciences, Mohammed V University, Rabat, Morocco; b Department of Biochemistry, Faculty of Education & Science, AlBaydha University, Yemen; c KU Leuven, Chemistry Department, Celestijnenlaan 200F box 2404, Leuven (Heverlee), B-3001, Belgium; d Laboratory of Medicinal Chemistry, Drug Sciences Research Center, Faculty of, Medicine and Pharmacy, Mohammed V University in Rabat, Morocco

**Keywords:** crystal structure, density functional theory, quinoxaline, hydrogen bond, Hirshfeld surface analysis

## Abstract

In the title mol­ecule, C_23_H_28_N_2_O, the phenyl ring is inclined to the quinoxaline ring system at a dihedral angle of 20.40 (9)°. In the crystal, C—H⋯O inter­actions between neighbouring mol­ecules form chains along the *a*-axis direction. Hirshfeld surface analysis indicates that the most important contributions to the crystal packing are from H⋯H (70.6%), H⋯C/C⋯H (15.5%) and H⋯O/O⋯H (4.6%) inter­actions. The optimized structure calculated using density functional theory at the B3LYP/6–311 G(d,p) level is compared with the experimentally determined structure in the solid state. The calculated highest occupied mol­ecular orbital (HOMO) and lowest unoccupied mol­ecular orbital (LUMO) energy gap is 3.8904 eV. Part of the *n*-nonyl chain attached to one of the nitro­gen atoms of the quinoxaline ring system shows disorder and was refined with a double conformation with occupancies of 0.604 (11) and 0.396 (11).

## Abstract 2

The phenyl-quinoxaline moiety in the title compound is not planar. In the crystal, C—H⋯O hydrogen bonds between neighboring quinoxaline rings form chains along the *a* axis direction.

## Chemical context   

Nitro­gen-based structures have attracted increased attention in structural and inorganic chemistry in recent years because of their inter­esting properties (Chkirate *et al.*, 2019 ▸, 2020*a* ▸,*b* ▸, 2021 ▸, 2022 ▸; Bouzian *et al.*, 2021 ▸). The family of quinoxalines, particularly those containing the quinoxalin-2-one moiety, is important in medicinal chemistry because of their wide range of pharmacological applications, including their use as anti-tumor active agents (Galal *et al.*, 2014 ▸), and their anti­microbial (Carta *et al.*, 2003 ▸) and biological (Carta *et al.*, 2002 ▸) activity. In particular, 3-phenyl­quinoxaline derivatives are used as anti-cancer drugs (Abad, Sallam *et al.*, 2021 ▸). They also have anti-folate activities (Corona *et al.*, 2008 ▸). Given the wide range of therapeutic applications for such compounds, and in a contin­uation of the work already carried out on the synthesis of compounds resulting from quinoxalin-2-one (Al Ati *et al.*, 2021 ▸), a similar approach gave the title compound, 1-nonyl-3-phenyl­quinoxalin-2-one C_23_H_28_N_2_O, (I). Besides the synthesis, we also report the mol­ecular and crystal structures along with a Hirshfeld surface analysis and a density functional theory computational calculation carried out at the B3LYP/6–311G(d,p) level.
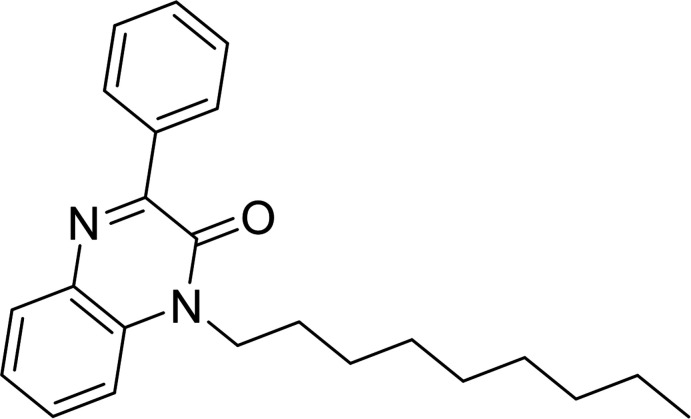


## Structural commentary   

**Figure 1:**
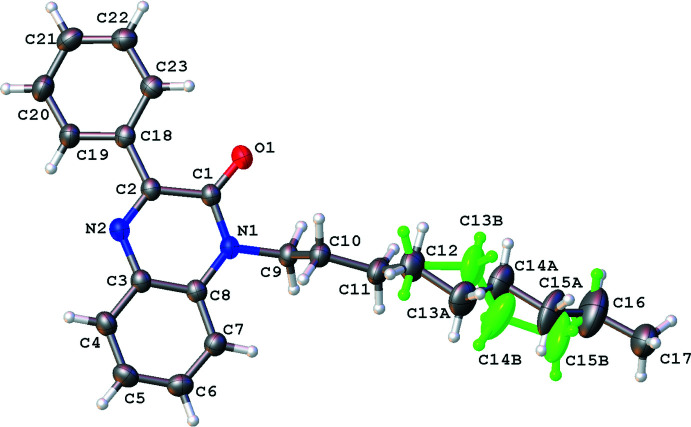
Mol­ecular structure of the title compound with the atom-labelling scheme and ellipsoids drawn at the 50% probability level. The disordered component of the *n*-nonyl chain with occupancy 0.396 (11) is shown in green.

The title compound crystallizes in the triclinic space group *P* 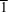 with one mol­ecule in the asymmetric unit (Fig. 1 ▸). The mol­ecule is not planar, as indicated by the torsion angles C1—C2—C18—C23 [−18.6 (3)°] and N2—C2—C18—C19 [−17.3 (3)°]. The best plane of the phenyl ring C18–C23 (r.m.s. deviation = 0.006 Å) makes a dihedral angle of 20.40 (9)° with the best plane of the quinoxaline ring system N1/C1/C2/N2/C3–C8 (r.m.s. deviation = 0.029 Å). This allows two intra­molecular inter­actions C23—H23⋯O1 and C19—H19⋯N2 (Table 1 ▸). The *n*-nonyl chain attached to one of the nitro­gen atoms of the quinoxaline ring system shows disorder and was refined with a double conformation for atoms C13 to C16 with occupancies of 0.604 (11) for C12*A*–C16*A* and 0.396 (11) for C12*B*–C16*B*. The *n*-nonyl chain of set *A* (starting from C9) has a *ap*, *ap*, *ap*, +*sc*, *ap*, *ap*, *ap* conformation, while for set *B* the conformation can be describes as *ap*, *ap*, *ap*, −*sc*, *ap*, −*sc*, *ap*.

## Supra­molecular features and Hirshfeld surface analysis   

**Figure 2:**
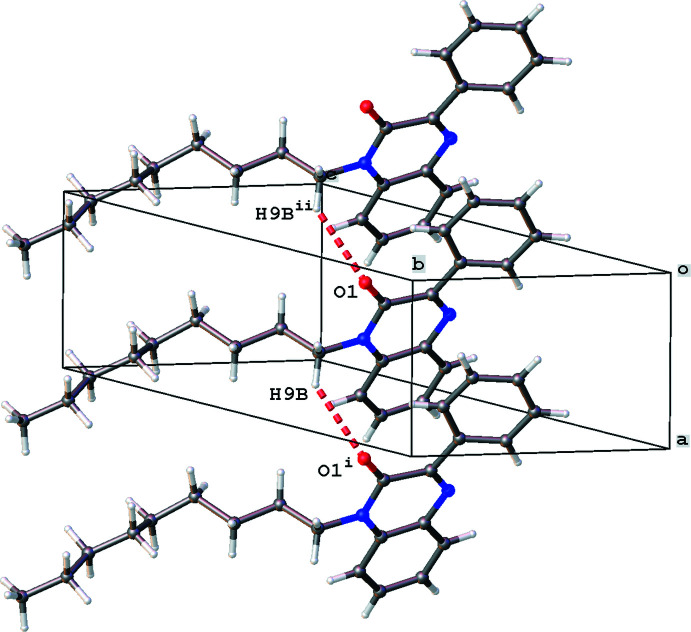
Partial view of the crystal packing of the title compound showing the C—H⋯O inter­action (red dashed lines) and chain formation in the *a-*axis direction. Only the major component of the *n*-nonyl chain is shown. Symmetry codes: (i) 1 + *x*, *y*, *z*; (ii) −1 + *x*, *y*, *z*.

**Figure 3:**
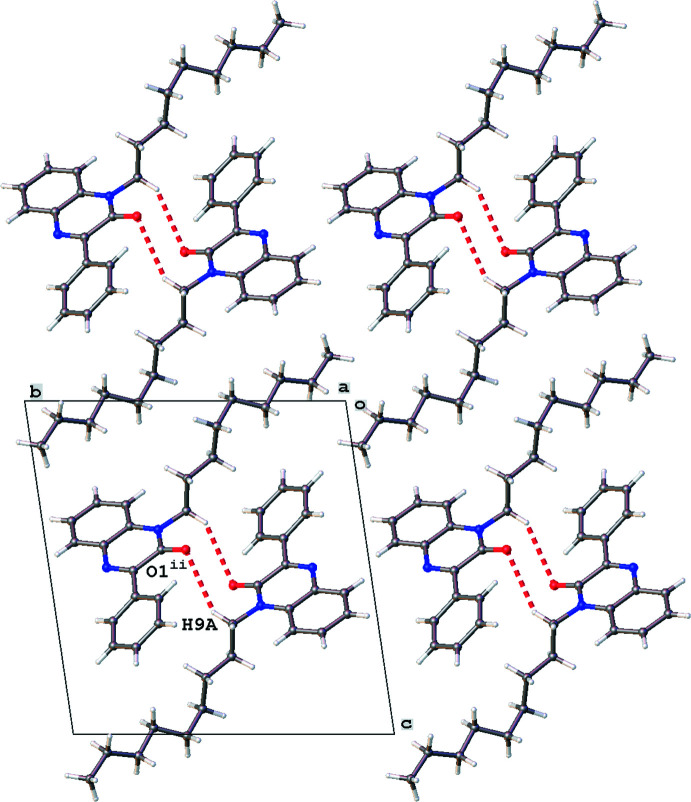
A view down the *a* axis of the crystal packing of the title compound showing the alternating layers of *n*-octyl chains and aromatic rings. Only the major disorder component of the *n*-nonyl chain is shown.

The crystal packing is characterized by C9—H9*B*⋯O1 inter­actions [Fig. 2 ▸; H9*B*⋯O1^i^ = 2.772 Å; symmetry code: (i) 1 + *x*, *y*, *z*] resulting in ribbon formation in the *a*-axis direction. Parallel ribbons show short C9—H9*A*⋯O1 contacts [Fig. 3 ▸; H9*A*⋯O1^ii^ = 2.899 Å; symmetry code: (ii) 1 − *x*, 1 − *y*, 1 − *z*]. The crystal packing shows layers of *n*-nonyl chains parallel to the (110) plane with layers of rings in between. Despite the presence of aromatic rings, the packing shows no C—H⋯π or π–π inter­actions [the shortest centroid–centroid distance is 3.8945 (15) Å for rings N1/N2/C1–C3/C8 and C18–C23]. The unit cell contains no residual solvent-accessible voids.

**Figure 4:**
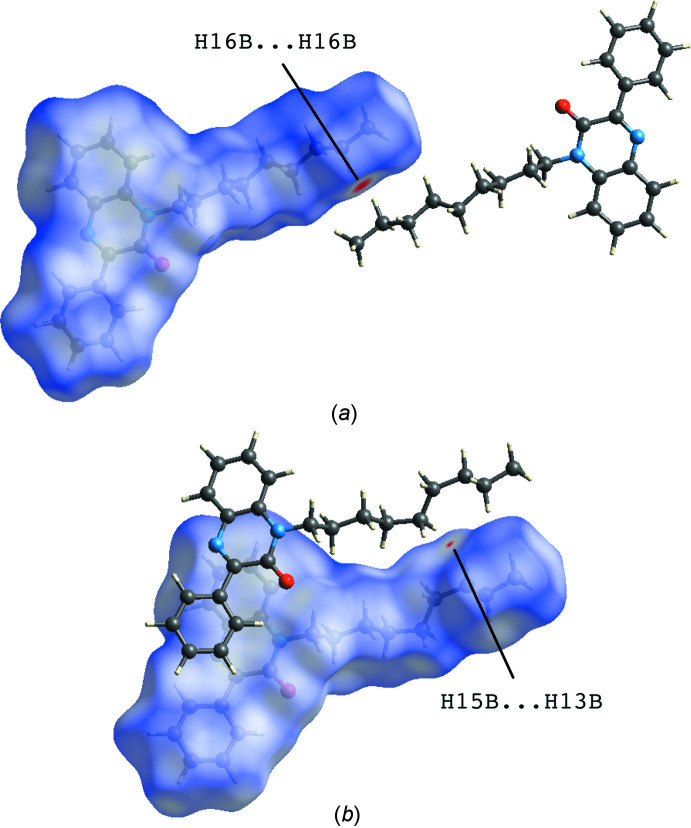
View of the three-dimensional Hirshfeld surface plotted over *d*_norm_ for (*a*) the major component (range −0.3582 to 1.3718 a.u.) and (*b*) the minor component (range −0.0395 to 1.5398 a.u.) of the title compound.

The *CrystalExplorer* program (Turner *et al.*, 2017 ▸) was used to further investigate and visualize the inter­molecular inter­actions of (I). The Hirshfeld surfaces for the major and minor occupancy components plotted over *d*_norm_ are shown in Fig. 4 ▸. The Hirshfeld surface of the major component (Fig. 4 ▸ *a*) is dominated by white regions representing contacts equal to the van der Waals separation and shows only one red spot (close contacts with a negative *d*_norm_ value) indicative of a H16*B*⋯H16*B* ^iii^ contact [1.995 Å; symmetry code: (iii) 2 − *x*, 2 − *y*, 2 − *z*]. A similar observation is made for the minor component (Fig. 4 ▸ *b*) where the tiny red spot represents a H15*B*⋯H13*B* ^i^ contact (2.316 Å).

**Figure 5:**
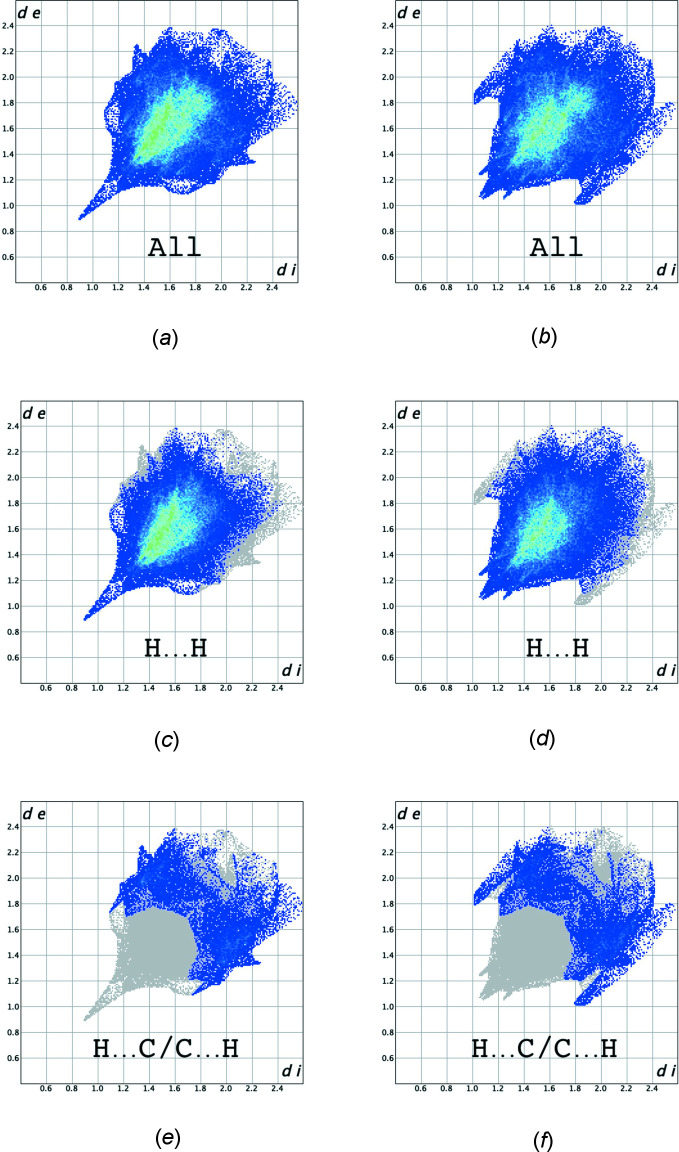
The full two-dimensional fingerprint plots showing (*a*,*b*) all inter­actions, and delineated into (*c*,*d*) H⋯H and (*e*,*f*) H⋯C/C⋯H inter­actions for the major (left) and minor (right) component of the title compound. The *d*_i_ and *d*_e_ values are the closest inter­nal and external distances (in Å) from points on the Hirshfeld surface.

The overall two-dimensional fingerprint plots (McKinnon *et al.*, 2007 ▸) for the two components are shown in Fig. 5 ▸ *a* and *b*, while those delineated into H⋯H and H⋯C/C⋯H contacts are illustrated in Fig. 5 ▸ *c*–*f*, respectively, together with their relative contributions to the Hirshfeld surface. The most important inter­action is H⋯H, contributing 70.6% (major component) or 70.5% (minor component) to the overall crystal packing, which is reflected in Fig. 5 ▸ *c* and *d* as widely scattered points of high density due to the large hydrogen content of the mol­ecule, with a sharp tip at *d*_e_ = *d*_i_ = 0.87 Å in the case of the major component. The second most important are C—H inter­actions, contributing 15.5% (major component) or 15.6% (minor component), for which the fingerprint plot (Fig. 5 ▸ *e* and *f*) shows characteristic wings with tips at *d_e_* + *d*_i_ ≃ 2.80 Å. Other contacts contribute only 4.6% (H⋯O/O⋯H), 4.3% (C⋯C), 2.4% (H⋯N/N⋯H), 2.2% (N⋯C/C⋯N), 0.3% (O⋯O) and 0.1% (O⋯C/C⋯O) to the Hirshfeld surface.

## Density functional theory calculations   

**Figure 6:**
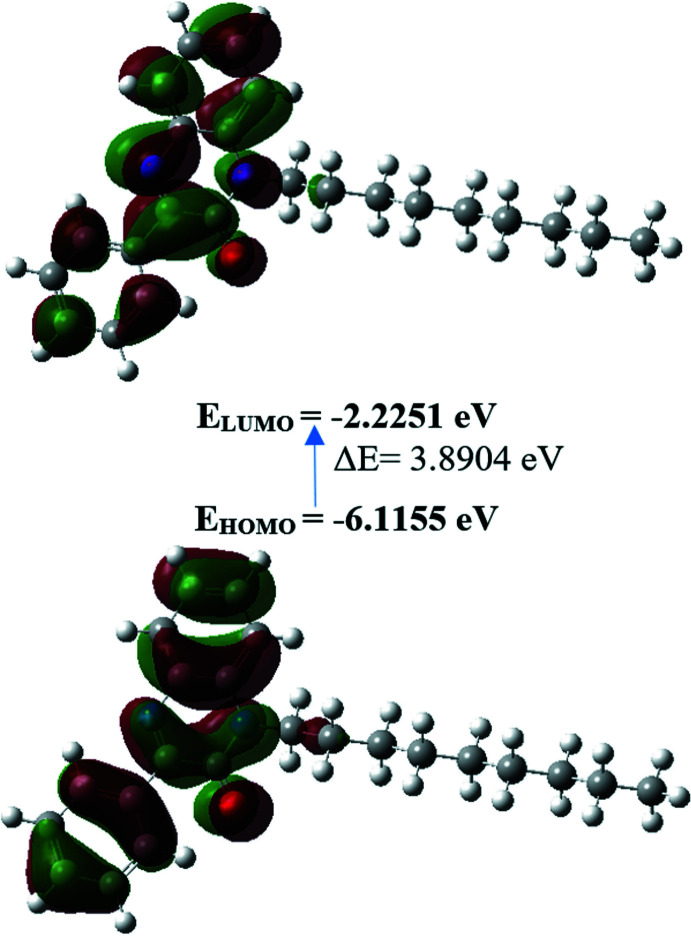
HOMO-LUMO and the energy band gap of the title compound.

The structure in the gas phase of the title compound was optimized by means of density functional theory. The density functional theory calculation was performed by the hybrid B3LYP method and the 6–311 G(d,p) basis-set, which is based on Becke’s model (Becke, 1993 ▸) and considers a mixture of the exact (Hartree–Fock) and density functional theory exchange utilizing the B3 functional, together with the LYP correlation functional (Lee *et al.*, 1988 ▸). After obtaining the converged geometry, the harmonic vibrational frequencies were calculated at the same theoretical level to confirm that the number of imaginary frequencies is zero for the stationary point. Both the geometry optimization and harmonic vibrational frequency analysis of the title compound were performed with the *GAUSSIAN 09* program (Frisch *et al.*, 2009 ▸). Theoretical and experimental results related to bond lengths and angles, which are in good agreement, are summarized in Table 2 ▸. Calculated numerical values for the title compound, including electronegativity (*χ*), hardness (*η*), ionization potential (*I*), dipole moment (*μ*), electron affinity (*A*), electrophilicity (*ω*) and softness (*σ*), are collated in Table 3 ▸. The electron transition from the highest occupied mol­ecular orbital (HOMO) to the lowest unoccupied mol­ecular orbital (LUMO) energy level is shown in Fig. 6 ▸. The HOMO and LUMO are localized in the plane extending over the whole 1-nonyl-3-phenyl­quinoxalin-2-one system. The energy band gap [*ΔE* = *E*_LUMO_ − *E*_HOMO_] of the mol­ecule is 3.8904 eV, and the frontier mol­ecular orbital energies, *E*_HOMO_ and *E*_LUMO_, are −6.1155 and −2.2251 eV, respectively.

## Database survey   

**Figure 7:**
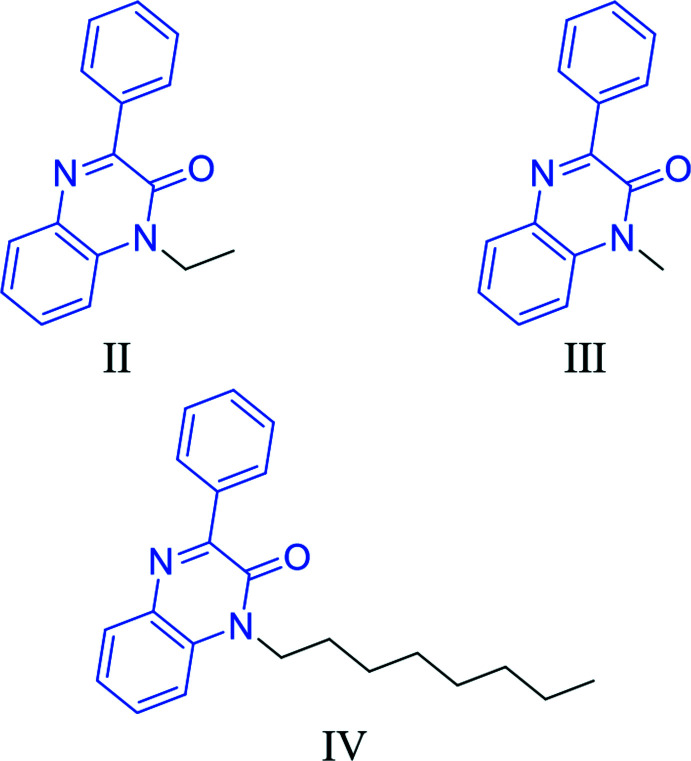
Structures similar to (I): (II) (CSD refcode MAGBIJ), (III) (CSD refcode BUDMAP) and (IV) (CSD refcode ASAZEC) obtained during the database search. The search fragment is indicated in blue.

A search of the Cambridge Structural Database (CSD version 5.42, updated May 2021; Groom *et al.*, 2016 ▸) for the quinoxalin-2(1*H*)-one fragment yielded multiple matches (180 hits). Of these, three compounds had an alkyl substituent on N1 and a phenyl ring on C2 comparable to (I) and are shown in Fig. 7 ▸. The first two compounds carry an ethyl [(II), refcode MAGBIJ; Al Ati *et al.*, 2021 ▸] or methyl [(III), refcode BUDMAP; Benzeid *et al.*, 2009 ▸] on N1. The third one [(IV), refcode ASAZEC; Abad, Ferfra *et al.*, 2021 ▸] has an *n*-octyl chain on N1 instead of a *n*-nonyl chain. The phenyl ring in MAGBIJ is inclined to the quinoxaline ring system by 25.81 (12)°. For BUDMAP, the dihedral angles are 19.3 (1) and 30.4 (1)° for the two mol­ecules present in the asymmetric unit. For ASAZEC, the dihedral angle is 12.90 (4)° and no disorder is observed in the *n*-octyl chain, which could be the consequence of the data collection being undertaken at 150 (2) K. Despite the similarity to the title compound, ASAZEC crystallizes in space group *C*2/*c* and the mol­ecules are linked by C—H⋯π inter­actions and form stacks in the *b*-axis direction.

## Synthesis and crystallization   

To a solution of 3-phenyl­quinoxalin-2(1*H*)-one (0.5 g, 2.25 mmol) in di­chloro­methane (20 ml) were added 1-chloro­nonane (0.2 ml, 2.25 mmol), sodium hydroxide (0.1 g, 2.25 mmol) and a catalytic qu­antity of tetra-*n*-butyl­ammonium bromide. The reaction mixture was stirred at room temperature for 24 h. The solution was filtered and the solvent removed under reduced pressure. The residue thus obtained was chromatographed on a silica gel column using a hexa­ne/ethyl acetate 9:1 mixture as eluent. The solid obtained was recrystallized from ethanol to afford colourless crystals (yield: 70%). ^1^H NMR (300 MHz, CDCl_3_) δ ppm: 0.89 (*t*, 3H, CH_3_, *J* = 6 Hz); 1.19–1.42 (*m*, 12H, CH_2_); 1.65–1.76 (quin, 2H, N—CH_2_—CH_2_); 4.20 (*t*, 2H, N—CH_2_, *J* = 6 Hz); 7.22–8.24 (*m*, 9H, CH_arom_); ^13^C NMR (75 MHz, CDCl_3_) δ ppm: 14.12 (CH_3_); 22.67, 27.11, 27.32, 29.24, 29.36, 29.51, 31.85 (CH_2_); 42.68 (N—CH_2_); 113.59, 123.49, 128.05, 129.63, 130.22, 130.28, 130.72 (CH_arom_); 132.61, 133.42, 136.14, 154.11 (C_q_); 154.40 (C=O).

## Refinement   

Crystal data, data collection and structure refinement details are given in Table 4 ▸. C-bound H atoms were positioned geometrically (C—H = 0.93–0.97 Å) and included as riding contributions with isotropic displacement parameters fixed at 1.2 times *U*_eq_ of the parent atoms (1.5 for methyl groups). During the refinement, the difference-Fourier map revealed disorder for atoms C13, C14 and C15 of the nonyl chain and two conformations were refined with distance restraints (1.512 Å) for the C—C bonds involved and RIGU restraints for the nonyl chain C11–C17. At the end of the refinement, the occupancy factors of the two components converged to 0.604 (11) and 0.396 (11) and the final difference-Fourier map showed no residual peaks of chemical significance.

## Supplementary Material

Crystal structure: contains datablock(s) I. DOI: 10.1107/S2056989021009737/dj2034sup1.cif

Structure factors: contains datablock(s) I. DOI: 10.1107/S2056989021009737/dj2034Isup3.hkl

Supporting information file. DOI: 10.1107/S2056989021009737/dj2034Isup3.cml

CCDC reference: 2110486

Additional supporting information:  crystallographic information; 3D view; checkCIF report

## Figures and Tables

**Table 1:** Hydrogen-bond geometry (Å, °)

*D*—H⋯*A*	*D*—H	H⋯*A*	*D*⋯*A*	*D*—H⋯*A*
C19—H19⋯N2	0.93	2.44	2.758 (3)	100
C23—H23⋯O1	0.93	2.21	2.832 (3)	123

**Table 2:** Comparison (X-ray and density functional theory) of selected bond lengths and angles (Å, °)

	X-ray	B3LYP/6–311G(d,p)
O1—C1	1.221 (3)	1.2236
N1—C1	1.379 (3)	1.3975
N1—C8	1.387 (3)	1.3892
N1—C9	1.474 (3)	1.4735
N2—C2	1.296 (3)	1.299
N2—C3	1.384 (3)	1.3723
C2—C18	1.481 (3)	1.4862
C1—N1—C8	122.74 (19)	122.5778
C1—N1—C9	116.64 (19)	116.1328
C8—N1—C9	120.60 (19)	121.2682
O1—C1—N1	120.6 (2)	120.2255
O1—C1—C2	124.1 (2)	124.5602
N1—C1—C2	115.22 (19)	115.2104
C2—N2—C3	120.3 (2)	120.949
N2—C2—C1	122.0 (2)	121.844
N2—C2—C18	117.6 (2)	117.4937
N2—C3—C4	118.7 (2)	118.5343
N2—C3—C8	121.6 (2)	121.9008
N1—C8—C3	117.6 (2)	117.4153
N1—C8—C7	123.5 (2)	123.4308
N1—C9—C10	112.61 (19)	112.9655

**Table 3:** Calculated energies

Mol­ecular Energy	Compound (I)
Total Energy, *TE* (eV)	−29343.5617
*E*_HOMO_ (eV)	−6.1155
*E*_LUMO_ (eV)	−2.2251
Gap, *ΔE* (eV)	3.8904
Dipole moment, *μ* (Debye)	3.0783
Ionization potential, *I* (eV)	6.1155
Electron affinity, *A*	2.2251
Electronegativity, *χ*	4.1703
Hardness, *η*	1.9452
Electrophilicity index, *ω*	4.4703
Softness, *σ*	0.5141
Fraction of electron transferred, *ΔN*	0.7274

**Table 4:** Experimental details

Crystal data	
Chemical formula	C_23_H_28_N_2_O
*M*_r_	348.47
Crystal system, space group	Triclinic, *P*\overline{1}
Temperature (K)	293
*a*, *b*, *c* (Å)	5.2353 (2), 13.5065 (5), 14.3158 (5)
α, β, γ (°)	98.045 (3), 98.327 (3), 91.255 (3)
*V* (Å^3^)	990.83 (6)
*Z*	2
Radiation type	Mo *K*α
μ (mm^−1^)	0.07
Crystal size (mm)	0.45 × 0.3 × 0.15

Data collection	
Diffractometer	SuperNova, Single source at offset/far, Eos
Absorption correction	Multi-scan (*CrysAlis PRO*; Rigaku OD, 2018 ▸)
*T*_min_, *T*_max_	0.686, 1.000
No. of measured, independent and observed [*I* > 2σ(*I*)] reflections	20242, 4058, 2864
*R*_int_	0.022
(sin θ/λ)_max_ (Å^−1^)	0.625

Refinement	
*R*[*F*^2^ > 2σ(*F* ^2^)], *wR*(*F* ^2^), *S*	0.070, 0.240, 1.05
No. of reflections	4058
No. of parameters	264
No. of restraints	70
H-atom treatment	H-atom parameters constrained
Δρ_max_, Δρ_min_ (e Å^−3^)	0.37, −0.45

Computer programs: *CrysAlis PRO* (Rigaku OD, 2018 ▸), *SHELXT2014/5* (Sheldrick, 2015*a* ▸), *SHELXL2016/4* (Sheldrick, 2015*b* ▸) and *OLEX2* (Dolomanov *et al.*, 2009 ▸).

## supplementary crystallographic information

### Crystal data

**Table:**

C_23_H_28_N_2_O	*Z* = 2
*M**_r_* = 348.47	*F*(000) = 376
Triclinic, *P*1	*D*_x_ = 1.168 Mg m^−^^3^
*a* = 5.2353 (2) Å	Mo *K*α radiation, λ = 0.71073 Å
*b* = 13.5065 (5) Å	Cell parameters from 6605 reflections
*c* = 14.3158 (5) Å	θ = 3.0–26.4°
α = 98.045 (3)°	µ = 0.07 mm^−^^1^
β = 98.327 (3)°	*T* = 293 K
γ = 91.255 (3)°	Block, colourless
*V* = 990.83 (6) Å^3^	0.45 × 0.3 × 0.15 mm

### Data collection

**Table:**

SuperNova, Single source at offset/far, Eos diffractometer	4058 independent reflections
Radiation source: micro-focus sealed X-ray tube, SuperNova (Mo) X-ray Source	2864 reflections with *I* > 2σ(*I*)
Mirror monochromator	*R*_int_ = 0.022
Detector resolution: 15.9631 pixels mm^-1^	θ_max_ = 26.4°, θ_min_ = 2.9°
ω scans	*h* = −6→6
Absorption correction: multi-scan (CrysAlisPro; Rigaku OD, 2018)	*k* = −16→16
*T*_min_ = 0.686, *T*_max_ = 1.000	*l* = −17→17
20242 measured reflections	

### Refinement

**Table:**

Refinement on *F*^2^	Primary atom site location: dual
Least-squares matrix: full	Hydrogen site location: inferred from neighbouring sites
*R*[*F*^2^ > 2σ(*F*^2^)] = 0.070	H-atom parameters constrained
*wR*(*F*^2^) = 0.240	*w* = 1/[σ^2^(*F*_o_^2^) + (0.1119*P*)^2^ + 0.4832*P*] where *P* = (*F*_o_^2^ + 2*F*_c_^2^)/3
*S* = 1.05	(Δ/σ)_max_ < 0.001
4058 reflections	Δρ_max_ = 0.37 e Å^−^^3^
264 parameters	Δρ_min_ = −0.45 e Å^−^^3^
70 restraints	

### Special details

**Table:**

Geometry. All esds (except the esd in the dihedral angle between two l.s. planes) are estimated using the full covariance matrix. The cell esds are taken into account individually in the estimation of esds in distances, angles and torsion angles; correlations between esds in cell parameters are only used when they are defined by crystal symmetry. An approximate (isotropic) treatment of cell esds is used for estimating esds involving l.s. planes.

### Fractional atomic coordinates and isotropic or equivalent isotropic displacement parameters (Å^2^)

**Table:**

	*x*	*y*	*z*	*U*_iso_*/*U*_eq_	Occ. (<1)
O1	0.3177 (4)	0.43358 (13)	0.55347 (14)	0.0685 (5)	
N1	0.6712 (4)	0.35716 (13)	0.61382 (14)	0.0498 (5)	
C1	0.4408 (5)	0.35784 (17)	0.55355 (17)	0.0510 (5)	
N2	0.4810 (4)	0.17973 (14)	0.50005 (14)	0.0527 (5)	
C2	0.3592 (4)	0.26106 (16)	0.49062 (16)	0.0489 (5)	
C3	0.6998 (5)	0.18102 (17)	0.56737 (17)	0.0516 (6)	
C4	0.8245 (5)	0.09152 (19)	0.5763 (2)	0.0625 (7)	
H4	0.755288	0.032153	0.539594	0.075*	
C5	1.0489 (6)	0.0911 (2)	0.6392 (2)	0.0698 (7)	
H5	1.131744	0.031553	0.644889	0.084*	
C6	1.1516 (5)	0.1794 (2)	0.6940 (2)	0.0672 (7)	
H6	1.305232	0.178777	0.735651	0.081*	
C7	1.0310 (5)	0.2677 (2)	0.68807 (18)	0.0594 (6)	
H7	1.100928	0.326025	0.726530	0.071*	
C8	0.8032 (4)	0.27050 (17)	0.62436 (16)	0.0497 (5)	
C9	0.7704 (5)	0.45374 (17)	0.66992 (17)	0.0547 (6)	
H9A	0.711702	0.507452	0.634775	0.066*	
H9B	0.957721	0.455955	0.678226	0.066*	
C10	0.6832 (5)	0.47073 (19)	0.76690 (18)	0.0606 (6)	
H10A	0.498084	0.479028	0.758694	0.073*	
H10B	0.717777	0.412121	0.798120	0.073*	
C11	0.8169 (6)	0.5614 (2)	0.8298 (2)	0.0745 (8)	
H11A	0.798334	0.618437	0.795459	0.089*	
H11B	0.999863	0.549819	0.843410	0.089*	
C12	0.7119 (8)	0.5865 (3)	0.9237 (2)	0.0978 (11)	
H12C	0.774577	0.539337	0.966060	0.117*	0.396 (11)
H12D	0.524815	0.579172	0.911703	0.117*	0.396 (11)
H12A	0.555095	0.622546	0.911469	0.117*	0.604 (11)
H12B	0.663609	0.524143	0.944183	0.117*	0.604 (11)
C13B	0.791 (3)	0.6928 (9)	0.9730 (12)	0.143 (6)	0.396 (11)
H13A	0.733512	0.738151	0.928155	0.172*	0.396 (11)
H13B	0.694988	0.706615	1.026028	0.172*	0.396 (11)
C14B	1.067 (3)	0.7193 (11)	1.0101 (13)	0.178 (8)	0.396 (11)
H14A	1.144535	0.724151	0.953271	0.214*	0.396 (11)
H14B	1.131999	0.659083	1.033095	0.214*	0.396 (11)
C15B	1.203 (5)	0.8041 (10)	1.0834 (14)	0.213 (9)	0.396 (11)
H15A	1.115971	0.809553	1.139137	0.255*	0.396 (11)
H15B	1.377951	0.784635	1.103084	0.255*	0.396 (11)
C13A	0.8860 (18)	0.6470 (6)	1.0052 (4)	0.108 (3)	0.604 (11)
H13C	0.803887	0.653364	1.062129	0.129*	0.604 (11)
H13D	1.046676	0.613550	1.018024	0.129*	0.604 (11)
C14A	0.9394 (19)	0.7469 (5)	0.9806 (5)	0.109 (3)	0.604 (11)
H14C	0.778583	0.778877	0.963392	0.130*	0.604 (11)
H14D	1.033828	0.741313	0.926736	0.130*	0.604 (11)
C15A	1.095 (3)	0.8071 (7)	1.0650 (5)	0.151 (4)	0.604 (11)
H15C	0.983493	0.820608	1.113487	0.182*	0.604 (11)
H15D	1.229528	0.765333	1.089666	0.182*	0.604 (11)
C16	1.2192 (15)	0.9031 (5)	1.0549 (5)	0.208 (3)	
H16C	1.047455	0.927886	1.040740	0.250*	0.396 (11)
H16D	1.302078	0.900230	0.998269	0.250*	0.396 (11)
H16A	1.331550	0.888114	1.006973	0.250*	0.604 (11)
H16B	1.083193	0.942764	1.027665	0.250*	0.604 (11)
C17	1.3710 (9)	0.9691 (3)	1.1345 (4)	0.1294 (16)	
H17A	1.353016	1.037431	1.124155	0.194*	
H17B	1.549689	0.953042	1.138569	0.194*	
H17C	1.309895	0.959940	1.192911	0.194*	
C18	0.1379 (5)	0.25620 (17)	0.41263 (17)	0.0517 (6)	
C19	0.1175 (6)	0.1751 (2)	0.33936 (19)	0.0663 (7)	
H19	0.242243	0.127165	0.340750	0.080*	
C20	−0.0844 (6)	0.1652 (2)	0.2653 (2)	0.0780 (8)	
H20	−0.094842	0.110612	0.217213	0.094*	
C21	−0.2709 (6)	0.2351 (2)	0.2615 (2)	0.0749 (8)	
H21	−0.408785	0.227544	0.211843	0.090*	
C22	−0.2513 (5)	0.3164 (2)	0.3319 (2)	0.0680 (7)	
H22	−0.375143	0.364646	0.328908	0.082*	
C23	−0.0503 (5)	0.32754 (19)	0.40714 (19)	0.0574 (6)	
H23	−0.040380	0.382880	0.454350	0.069*	

### Atomic displacement parameters (Å^2^)

**Table:**

	*U*^11^	*U*^22^	*U*^33^	*U*^12^	*U*^13^	*U*^23^
O1	0.0726 (11)	0.0453 (9)	0.0818 (13)	0.0079 (8)	0.0004 (9)	−0.0005 (8)
N1	0.0560 (11)	0.0410 (10)	0.0511 (11)	−0.0013 (8)	0.0092 (9)	0.0018 (8)
C1	0.0567 (13)	0.0449 (12)	0.0511 (13)	0.0017 (10)	0.0094 (10)	0.0046 (10)
N2	0.0590 (11)	0.0431 (10)	0.0556 (11)	0.0006 (8)	0.0100 (9)	0.0046 (8)
C2	0.0544 (12)	0.0427 (11)	0.0506 (12)	−0.0014 (9)	0.0124 (10)	0.0058 (9)
C3	0.0596 (13)	0.0458 (12)	0.0503 (13)	0.0015 (10)	0.0110 (10)	0.0071 (10)
C4	0.0740 (16)	0.0466 (13)	0.0672 (16)	0.0068 (12)	0.0115 (13)	0.0079 (11)
C5	0.0749 (17)	0.0602 (16)	0.0772 (18)	0.0160 (13)	0.0096 (14)	0.0203 (14)
C6	0.0635 (15)	0.0762 (18)	0.0627 (16)	0.0087 (13)	0.0034 (12)	0.0179 (13)
C7	0.0627 (14)	0.0593 (15)	0.0545 (14)	0.0002 (11)	0.0060 (11)	0.0058 (11)
C8	0.0567 (13)	0.0466 (12)	0.0480 (12)	0.0014 (10)	0.0143 (10)	0.0083 (9)
C9	0.0591 (13)	0.0446 (12)	0.0574 (14)	−0.0047 (10)	0.0057 (11)	0.0014 (10)
C10	0.0643 (15)	0.0564 (14)	0.0583 (15)	−0.0017 (11)	0.0083 (12)	0.0000 (11)
C11	0.0707 (17)	0.0774 (18)	0.0667 (16)	−0.0048 (14)	0.0051 (13)	−0.0135 (14)
C12	0.110 (3)	0.101 (2)	0.075 (2)	0.001 (2)	0.0198 (18)	−0.0184 (18)
C13B	0.185 (12)	0.124 (9)	0.100 (11)	−0.017 (9)	0.017 (8)	−0.049 (8)
C14B	0.234 (15)	0.157 (11)	0.116 (11)	−0.097 (12)	−0.048 (11)	0.019 (9)
C15B	0.204 (19)	0.152 (8)	0.249 (18)	−0.094 (11)	0.037 (13)	−0.073 (8)
C13A	0.143 (6)	0.120 (5)	0.054 (3)	−0.002 (4)	0.009 (3)	−0.004 (3)
C14A	0.128 (7)	0.119 (5)	0.067 (4)	−0.024 (4)	0.009 (4)	−0.015 (3)
C15A	0.192 (11)	0.174 (7)	0.070 (4)	−0.062 (6)	0.028 (5)	−0.047 (4)
C16	0.213 (7)	0.174 (5)	0.206 (7)	−0.081 (5)	0.012 (5)	−0.051 (5)
C17	0.123 (3)	0.099 (3)	0.146 (4)	−0.008 (2)	−0.013 (3)	−0.015 (3)
C18	0.0560 (13)	0.0455 (12)	0.0544 (13)	−0.0054 (10)	0.0102 (10)	0.0089 (10)
C19	0.0733 (17)	0.0538 (14)	0.0655 (16)	0.0023 (12)	−0.0016 (13)	−0.0008 (12)
C20	0.086 (2)	0.0679 (18)	0.0694 (18)	−0.0039 (15)	−0.0073 (15)	−0.0054 (14)
C21	0.0684 (17)	0.081 (2)	0.0702 (18)	−0.0068 (15)	−0.0100 (14)	0.0153 (15)
C22	0.0617 (15)	0.0711 (17)	0.0718 (17)	0.0044 (13)	0.0051 (13)	0.0174 (14)
C23	0.0570 (13)	0.0554 (13)	0.0616 (14)	−0.0001 (11)	0.0122 (11)	0.0114 (11)

### Geometric parameters (Å, º)

**Table:**

O1—C1	1.221 (3)	C7—C8	1.397 (3)
C13Bb—H13A	0.9700	C9—H9A	0.9700
C13Bb—H13B	0.9700	C9—H9B	0.9700
C13Bb—C14B	1.482 (10)	C9—C10	1.512 (3)
C14Bb—H14A	0.9700	C10—H10A	0.9700
C14Bb—H14B	0.9700	C10—H10B	0.9700
C14Bb—C15B	1.527 (9)	C10—C11	1.507 (4)
C15Bb—H15A	0.9700	C11—H11A	0.9700
C15Bb—H15B	0.9700	C11—H11B	0.9700
C13Aa—H13C	0.9700	C11—C12	1.523 (4)
C13Aa—H13D	0.9700	C12—H12C	0.9700
C13Aa—C14A	1.472 (8)	C12—H12D	0.9700
C14Aa—H14C	0.9700	C12—H12A	0.9700
C14Aa—H14D	0.9700	C12—H12B	0.9700
C14Aa—C15A	1.481 (7)	C12—C13B	1.528 (9)
C15Aa—H15C	0.9700	C12—C13A	1.500 (6)
C15Aa—H15D	0.9700	C16—H16C	0.9700
C15Bb—C16	1.456 (10)	C16—H16D	0.9700
C15Aa—C16	1.473 (5)	C16—H16A	0.9700
N1—C1	1.379 (3)	C16—H16B	0.9700
N1—C8	1.387 (3)	C16—C17	1.466 (7)
N1—C9	1.474 (3)	C17—H17A	0.9600
C1—C2	1.496 (3)	C17—H17B	0.9600
N2—C2	1.296 (3)	C17—H17C	0.9600
N2—C3	1.384 (3)	C18—C19	1.397 (3)
C2—C18	1.481 (3)	C18—C23	1.395 (3)
C3—C4	1.399 (3)	C19—H19	0.9300
C3—C8	1.410 (3)	C19—C20	1.375 (4)
C4—H4	0.9300	C20—H20	0.9300
C4—C5	1.373 (4)	C20—C21	1.374 (4)
C5—H5	0.9300	C21—H21	0.9300
C5—C6	1.385 (4)	C21—C22	1.374 (4)
C6—H6	0.9300	C22—H22	0.9300
C6—C7	1.369 (4)	C22—C23	1.382 (4)
C7—H7	0.9300	C23—H23	0.9300
			
C1—N1—C8	122.74 (19)	C15Bb—C16—C17	107.4 (8)
C14Aa—C13Aa—C12	109.4 (6)	N1—C8—C7	123.5 (2)
C14Bb—C13Bb—C12	119.6 (10)	C7—C8—C3	118.9 (2)
C14Bb—C13Bb—H13A	107.4	N1—C9—H9A	109.1
H13Ab—C13Bb—H13B	106.9	N1—C9—H9B	109.1
C14Bb—C13Bb—H13B	107.4	N1—C9—C10	112.61 (19)
C13Bb—C14Bb—H14A	104.0	H9A—C9—H9B	107.8
C1—N1—C9	116.64 (19)	C10—C9—H9A	109.1
C8—N1—C9	120.60 (19)	C10—C9—H9B	109.1
O1—C1—N1	120.6 (2)	C9—C10—H10A	109.1
O1—C1—C2	124.1 (2)	C9—C10—H10B	109.1
C15Bb—C14Bb—H14A	104.0	H10A—C10—H10B	107.8
H14Ab—C14Bb—H14B	105.4	C11—C10—C9	112.5 (2)
C13Bb—C14Bb—H14B	104.0	C11—C10—H10A	109.1
C15Bb—C14Bb—H14B	104.0	C11—C10—H10B	109.1
C13Bb—C14Bb—C15B	133.0 (17)	C10—C11—H11A	108.9
C14Bb—C15Bb—H15A	107.9	C10—C11—H11B	108.9
C14Bb—C15Bb—H15B	107.9	C10—C11—C12	113.5 (3)
H15Ab—C15Bb—H15B	107.2	H11A—C11—H11B	107.7
N1—C1—C2	115.22 (19)	C12—C11—H11A	108.9
C2—N2—C3	120.3 (2)	C12—C11—H11B	108.9
N2—C2—C1	122.0 (2)	C11—C12—H12C	109.1
N2—C2—C18	117.6 (2)	C11—C12—H12D	109.1
C18—C2—C1	120.4 (2)	C11—C12—H12A	107.9
N2—C3—C4	118.7 (2)	C11—C12—H12B	107.9
N2—C3—C8	121.6 (2)	C11—C12—C13B	112.6 (8)
C4—C3—C8	119.7 (2)	C17—C16—C15A	123.6 (7)
C3—C4—H4	119.9	C17—C16—H16C	110.2
C5—C4—C3	120.2 (2)	C12—C13Bb—H13A	107.4
C5—C4—H4	119.9	C12—C13Bb—H13B	107.4
C14Aa—C13Aa—H13C	109.8	C12—C13Aa—H13C	109.8
C14Aa—C13Aa—H13D	109.8	C12—C13Aa—H13D	109.8
H13Ca—C13Aa—H13D	108.2	C16—C15Bb—C14B	117.8 (12)
C13Aa—C14Aa—H14C	110.1	C17—C16—H16D	110.2
C15Aa—C14Aa—H14C	110.1	C17—C16—H16A	106.4
C15Aa—C14Aa—H14D	110.1	C17—C16—H16B	106.4
H14Ca—C14Aa—H14D	108.4	C16—C17—H17A	109.5
C13Aa—C14Aa—H14D	110.1	C16—C17—H17B	109.5
C13Aa—C14Aa—C15A	108.0 (6)	C16—C17—H17C	109.5
C14Aa—C15Aa—H15C	107.4	H17A—C17—H17B	109.5
C14Aa—C15Aa—H15D	107.4	H17A—C17—H17C	109.5
C4—C5—H5	120.1	H17B—C17—H17C	109.5
C4—C5—C6	119.9 (2)	C19—C18—C2	117.8 (2)
C6—C5—H5	120.1	C23—C18—C2	124.4 (2)
C5—C6—H6	119.4	C23—C18—C19	117.9 (2)
C7—C6—C5	121.2 (3)	C18—C19—H19	119.5
C7—C6—H6	119.4	C20—C19—C18	120.9 (3)
C6—C7—H7	119.9	C20—C19—H19	119.5
C6—C7—C8	120.2 (2)	C19—C20—H20	119.7
C8—C7—H7	119.9	C21—C20—C19	120.6 (3)
N1—C8—C3	117.6 (2)	C21—C20—H20	119.7
H15Ca—C15Aa—H15D	107.0	C20—C21—H21	120.4
C13Aa—C12—C11	117.4 (5)	C22—C21—C20	119.3 (3)
C13Bb—C12—H12C	109.1	C22—C21—H21	120.4
H12Cb—C12—H12D	107.8	C21—C22—H22	119.5
C13Bb—C12—H12D	109.1	C21—C22—C23	121.0 (3)
C13Aa—C12—H12A	107.9	C23—C22—H22	119.5
C13Aa—C12—H12B	107.9	C18—C23—H23	119.8
H12Aa—C12—H12B	107.2	C22—C23—C18	120.3 (3)
C15Bb—C16—H16C	110.2	C22—C23—H23	119.8
C15Bb—C16—H16D	110.2	C16—C15Bb—H15A	107.9
H16Cb—C16—H16D	108.5	C16—C15Bb—H15B	107.9
C15Aa—C16—H16A	106.4	C16—C15Aa—C14A	119.5 (7)
H16Aa—C16—H16B	106.5	C16—C15Aa—H15C	107.4
C15Aa—C16—H16B	106.4	C16—C15Aa—H15D	107.4
			
C13Aa—C14Aa—C15Aa—C16	168.0 (12)	C4—C3—C8—C7	0.7 (4)
C13Bb—C14Bb—C15Bb—C16	−76 (3)	C4—C5—C6—C7	1.1 (4)
C14Bb—C15Bb—C16—C17	−176.5 (18)	C5—C6—C7—C8	−1.4 (4)
C14Aa—C15Aa—C16—C17	178.4 (9)	C6—C7—C8—N1	−179.5 (2)
O1—C1—C2—N2	−172.5 (2)	C6—C7—C8—C3	0.5 (4)
O1—C1—C2—C18	9.4 (4)	C8—N1—C1—O1	172.5 (2)
N1—C1—C2—N2	7.6 (3)	C8—N1—C1—C2	−7.6 (3)
N1—C1—C2—C18	−170.52 (19)	C8—N1—C9—C10	−86.1 (3)
N1—C9—C10—C11	171.1 (2)	C8—C3—C4—C5	−1.1 (4)
C1—N1—C8—C3	2.8 (3)	C9—N1—C1—O1	−5.8 (3)
C1—N1—C8—C7	−177.2 (2)	C9—N1—C1—C2	174.13 (19)
C1—N1—C9—C10	92.3 (3)	C9—N1—C8—C3	−179.0 (2)
C1—C2—C18—C19	160.9 (2)	C9—N1—C8—C7	1.0 (3)
C1—C2—C18—C23	−18.6 (3)	C9—C10—C11—C12	173.8 (3)
N2—C2—C18—C19	−17.3 (3)	C10—C11—C12—C13Aa	157.2 (5)
N2—C2—C18—C23	163.2 (2)	C10—C11—C12—C13Bb	−163.5 (7)
N2—C3—C4—C5	176.9 (2)	C11—C12—C13Bb—C14Bb	−66.5 (19)
N2—C3—C8—N1	2.9 (3)	C11—C12—C13Aa—C14Aa	64.2 (9)
N2—C3—C8—C7	−177.2 (2)	C12—C13Aa—C14Aa—C15Aa	175.8 (8)
C2—N2—C3—C4	179.2 (2)	C12—C13Bb—C14Bb—C15Bb	−160.7 (14)
C2—N2—C3—C8	−2.8 (3)	C18—C19—C20—C21	0.1 (5)
C2—C18—C19—C20	179.3 (3)	C19—C18—C23—C22	1.0 (4)
C2—C18—C23—C22	−179.5 (2)	C19—C20—C21—C22	1.1 (5)
C3—N2—C2—C1	−2.5 (3)	C20—C21—C22—C23	−1.3 (5)
C3—N2—C2—C18	175.63 (19)	C21—C22—C23—C18	0.3 (4)
C3—C4—C5—C6	0.2 (4)	C23—C18—C19—C20	−1.2 (4)
C4—C3—C8—N1	−179.3 (2)		

### Hydrogen-bond geometry (Å, º)

**Table:**

*D*—H···*A*	*D*—H	H···*A*	*D*···*A*	*D*—H···*A*
C19—H19···N2	0.93	2.44	2.758 (3)	100
C23—H23···O1	0.93	2.21	2.832 (3)	123
